# Pulse width and intensity effects of pulsed electric fields on cancerous and normal skin cells

**DOI:** 10.1038/s41598-022-22874-x

**Published:** 2022-10-27

**Authors:** Xin Rao, Sophia Chen, Yasir Alfadhl, Xiaodong Chen, Lingling Sun, Liyang Yu, Jun Zhou

**Affiliations:** 1 School of Electronics and Information, Hangzhou Dianzi University, Hangzhou, 310018 China; 2 School of Medicine, Imperial College London, London, SW7 2AZ UK; 3 School of Electronic Engineering and Computer Science, Queen Mary University of London, London, E1 4NS UK; 4 School of Electronic Science and Engineering, University of Electronic Science and Technology of China, Chengdu, 610054 China

**Keywords:** Computational biophysics, Biomedical engineering

## Abstract

Microsecond pulsed electric fields (PEF) have previously been used for various tumour therapies, such as gene therapy, electrochemotherapy and irreversible electroporation (IRE), due to its demonstrated ability. However, recently nanosecond pulsed electric fields (nsPEF) have also been used as a potential tumor therapy via inducing cell apoptosis or immunogenic cell death to prevent recurrence and metastasis by interacting with intracellular organelles. A large proportion of the existing in-vitro studies of nsPEF on cells also suggests cell necrosis and swelling/blebbing can be induced, but the replicability and potential for other effects on cells suggesting a complicated process which requires further investigation. Therefore, this study investigated the effects of pulse width and intensity of nsPEF on the murine melanoma cells (B16) and normal murine fibroblast cells (L929) through electromagnetic simulation and in-vitro experiments. Through examining the evolution patterns of potential difference and electric fields on the intracellular compartments, the simulation has shown a differential effect of nsPEF on normal and cancerous skin cells, which explains well the results observed in the reported experiments. In addition, the modelling has provided a clear evidence that a few hundreds of ns PEF may have caused a mixed mode of effects, i.e. a ‘cocktail effect’, including cell electroporation and IRE due to an over their threshold voltage induced on the plasma membrane, as well as cell apoptosis and other biological effects caused by its interaction with the intracellular compartments. The in-vitro experiments in the pulse range of the hundreds of nanoseconds showed a possible differential cytotoxicity threshold of electric field intensity between B16 cells and L929 cells.

## Introduction

Pulsed electric fields (PEF) have been employed in many different types of tumor therapies, including gene therapy, electrochemotherapy and irreversible electroporation (IRE) due to the known advantages of being non-thermal and microinvasive^1–3^. The PEF with a duration of microseconds to milliseconds charges up the cell plasma membrane and once this voltage is above a certain threshold, hydrophilic pores are formed in the cell plasma membrane. The size of the pores is dependent on the field intensity and the size of the pores determines their reversibility. Hence, by regulating the field intensity of microsecond PEF, reversible electroporation (RE) and IRE can be stimulated precisely according to individual needs^4–6^.

Recent studies have shown that intense nanosecond pulsed electric fields (nsPEF) not only possess the advantages of traditional pulse electric fields as an independent physical therapy, but also can induce anti-cancer immunity in the treatment of local tumors to prevent recurrence and metastasis^7–11^. Instead of permeabilizing the plasma membrane in IRE or RE with microsecond PEF, nsPEF is thought to stimulate apoptosis, necroptosis, autophagy, and other biological effects by interacting with cytoskeleton, mitochondria, nuclear or other intracellular organelles^12–15^. However, a large proportion of the observations in the in-vitro studies involving nsPEF and cell interactions show cell necrosis and swelling/blebbing, implying this process is quite complicated^12,13,16^. Such findings of cell necrosis and swelling/blebbing suggestive of a compromised cell plasma membrane are predominantly shown with the use of nsPEF in the range of the hundreds of nanoseconds. The mixed modality of cell death and the range of biological effects caused by nsPEF stimulation with the pulse width of hundreds of nanoseconds, such as IRE, apoptosis, necroptosis and reversible electroporation is referred to as the ‘cocktail effect’. One approach to investigate this ‘cocktail effect’ is to prevent the conditions required for electroporation of the plasma membrane by reducing the pulse width of nsPEF from hundreds of nanoseconds to tens of nanoseconds^16–21^. Therefore, it is necessary to further investigate the pulse width and intensity effects of nsPEF on the cells.

On the other hand, the selective sensitivity of normal and cancerous cells to nsPEF is an important factor to be considered in treatment planning to improve the desired prognosis with minimal side effects. The relevant studies on cancerous and normal cell selective susceptibility to nsPEF are limited. Most of the studies focus on the cytotoxicity efficacy of specific PEF on different cancerous cells^17,19–24^. Yang et al. first reported differential sensitivities between malignant and normal skin cells—a basal cell carcinoma (BCC) cell line, and its sister normal cell line (TE) after exposure to nsPEF with a fixed pulse width of 30 ns and pulse intensity of 30 kV/cm, but with a varied pulse number. They reported a greater increase of caspase activation in the BCC cell line than the TE cell line and a larger decrease of cell viability in BCC cells than TE cells^23^. Gianulis et al. investigated the selective susceptibility of six cell lines (four cancerous cell lines and two normal cell lines) to nsPEF with a fixed pulse width of 300 ns and pulse intensity of 1.8 kV/cm, but again with a varied pulse number. They found that the cytotoxicity of six cell lines was all increased in three stages with the increase of pulse number^24^. However, they observed that the cytotoxic efficiency showed no apparent correlation with cell or nuclear size, cell morphology, metabolism level, or the extent of membrane disruption by nsPEF. Their observation is different from the result obtained in Yang et al.^23^, which found that cell viability is morphologically dependent. It is worth mentioning that the pulse width used in Gianulis et al. and Yang et al. is different, fixed at 300 ns and 30 ns respectively. Thus, this raises the question whether these different cell responses are related to the pulse width, the intensity, the number of pulses, or other pulse parameters, such as shape and rise/fall edges, which contribute to the frequency content of the pulses^25,26^.

In order to further study the pulse width and intensity effects and the selective sensitivity of cancerous and normal cells to nsPEF, we conducted electromagnetic simulation and in-vitro studies on murine melanoma B16 cells and murine fibroblast L929 cells by varying both pulse width and pulse intensity. The in-vitro experiments were conducted over a range of hundreds of nanoseconds by using a width tunable pulse generator developed in our research group^27^. Because of the increased cell division of cancerous cells, the cell size and nucleoplasmic ratio of cancerous cell are larger than those of normal cells according to the statistics^28,29^. Given there are significant morphological differences between cancerous and normal cells, the charging up time of their cellular compartment are different from each other’s, which leads to a different field distribution and provides possibility to achieving selective sensitivity for these two kinds of cells. Consequently, although the interaction between PEF and cells strictly depends on cell morphology has been studied^30–33^, it is necessary and essential to further investigate how these two kinds of cells respond to nsPEF over a wide range of pulse parameters.

In the first phase of our study, the multilayer physical models of cancerous cells and normal cells were developed to examine the potential difference evolution inside the cell when exposed to PEF with the voltage of 50–5 V across the electrodes, which is equivalent to the electric field strength of 20–2 kV/cm when only filled with the cell culture solution, for the pulse width of 30–100 μs^12–15,34–36^. Generally speaking, the types of cell models for this application can be classified into three categories: membrane aqueous pore model, passive equivalent circuit model and the physical model. The membrane aqueous pore model based on molecule simulation can show the response of the lipid bilayers under electric field for further understanding of the basic electroporation mechanism. However, limited by the current computing power, this model can only accommodate tens to hundreds thousands atoms at the nanoscale^37,38^. Thus, it considers only a small proportion of the membrane with pure lipid bilayers, which is not suitable for our study on cellular effect at the micron scale. The passive equivalent circuit model employs the equivalent resistors and capacitors to represent the cell compartments^39^ for observing the dynamic process of potential differences across the cell compartments. Though this model can predict the potential difference evolution pattern inside a cell, it is challenging to calculate the equivalent resistors and capacitors accurately for different compartments of the cell^40^. So, to observe the potential difference evolution on different intracellular compartments with reasonable computational load, the physical cell model is the only reasonable choice in this study^41,42^. Although the cell shape is simplified as spherical, the heterogeneous electrical properties and dimension of subcellular components are closely represented. The evolution of potential difference and electric field distribution inside the cell caused by the morphological difference and variable pulse width can be examined. The circuit models have also been adopted in our study to explain the results obtained in the physical models.

In the second phase, the cytotoxicity levels of murine melanoma B16 cells and murine fibroblast L929 cells treated by nsPEF with a pulse width of 300 and 500 ns and the electric field strength of 8–16 kV/cm were observed by flow cytometry fluorescence sorting (FACS). To control the number of study variables, other parameters of nsPEF were kept similar to those in other groups’ works^23,24,39,43^.

## Results and discussion

### Results of 2D cell modelling

**Figure 1:**
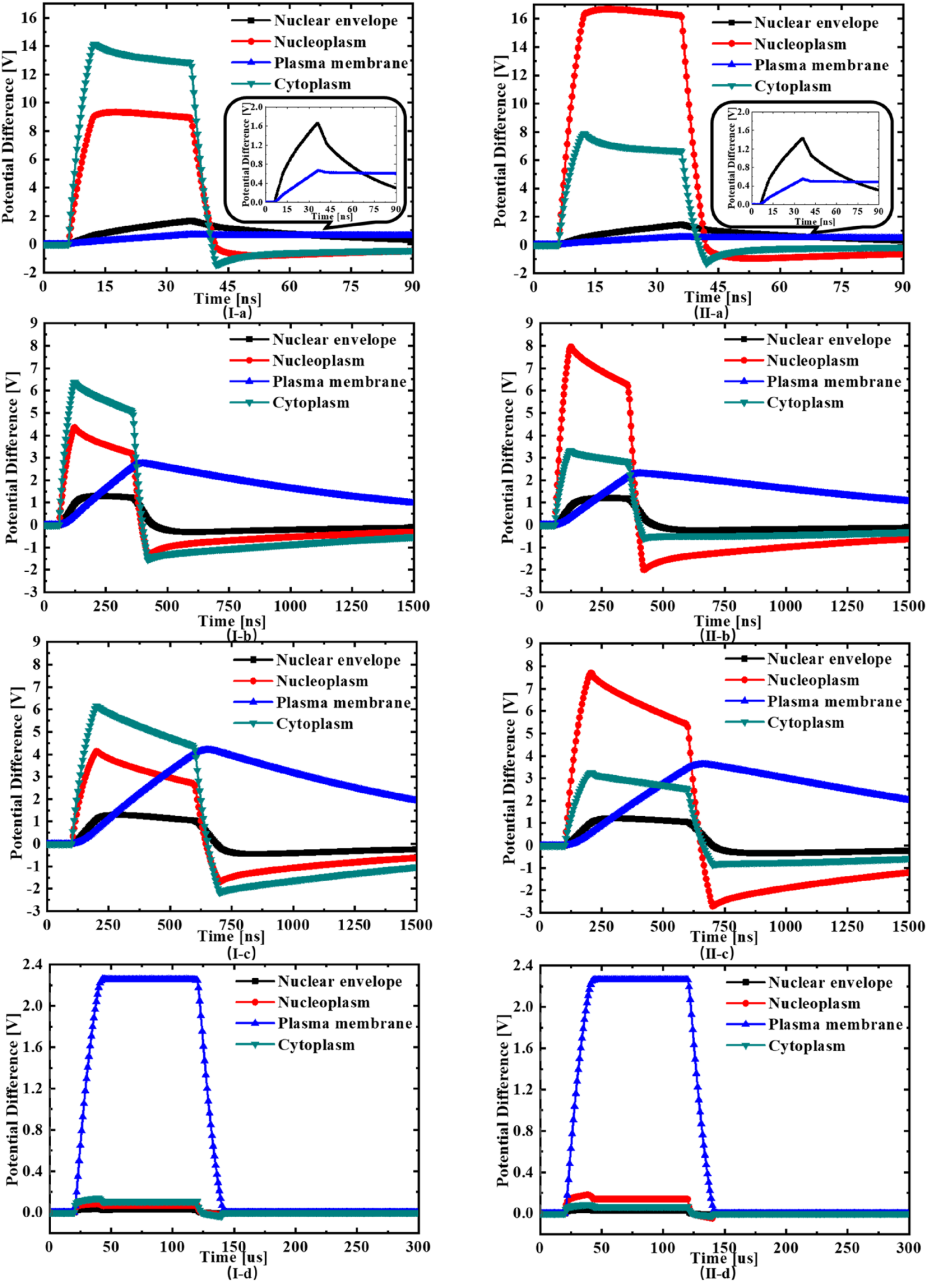
The simulated potential difference evolutions in the 2-D rotating models of the normal (**I-a**, **b**, **c**, **d**) and cancerous (**II-a**, **b**, **c**, **d**) cell suspended in a conductive solution between parallel electrodes biased with different time width pulses; (**I**, **II-a**): pulse width is 30 ns, voltage is 50 V; (**I**, **II-b**): pulse width is 300 ns , voltage is 25 V; (**I**, **II-c**): pulse width is 500 ns, voltage is 25 V; (**I**, **II-d**): pulse width is 100 μs, voltage is 5 V.

The evolution of the potential differences across the nuclear envelope (black curve), nucleoplasm (red curve), plasm membrane (blue curve), and cytoplasm (green curve) in normal and cancerous cells under different PEF are shown in Fig. 1. These potential differences are measured along the vertical central axis of the cell, which are also the maximum ones on each membrane or cell compartment^44^. The potential differences across the membrane and inner compartments are compared to show the pulse width and intensity effects of PEF.

As shown in Fig. 1I-a and II-a, when both cancerous and normal cells are stimulated with a PEF of 30 ns, the cytoplasm and the nucleoplasm in both cells are quickly charged up to a stable high potential difference. It is easy to understand this phenomenon since both cytoplasm and nucleoplasm have very small characteristic capacitance, and hence, a very short charging up time as shown in Table 1. The stable voltages are around 13 V and 9 V on the cytoplasm and the nucleoplasm of normal cell, respectively; while the stable voltages are around 16 V and 7 V on the nucleoplasm and the cytoplasm of cancerous cell, respectively.

**Table 1:** Characteristic capacitance and observed charging/discharging time constant.

	Characteristic capacitance	Observed time constant
**Plasma membrane**		
Cancerous cell	10.45 pF	1.2 μs
Normal cell	7.78 pF	1 μs
**Cytoplasm**		
Cancerous cell	0.09 pF	3.9 ns
Normal cell	0.03 pF	3.9 ns
**Nucleus envelope**		
Cancerous cell	2.09 pF	45.4 ns
Normal cell	0.61 pF	40 ns
**Nucleus cytoplasm**		
Cancerous cell	0.05 pF	6 ns
Normal cell	0.03 pF	5.7 ns

At the same time, the plasma membrane is hardly charged up during such a short pulse, with the potential differences across them being below 1 V because they have a large characteristic capacitance, thus need longer charging up time, as shown in Table 1. This set of results is in agreement with the current hypothesis that short nanosecond pulses only interact with the intracellular structures of the cell^45,46^. It is interesting to note that the nuclear envelope in both normal and cancerous cells is charged up with a rising potential difference well above 1 V (the corresponding electric field strengths are listed in Table 2) for a duration around 25 ns. It is generally accepted that the electroporation of membrane happens when the transmembrane potential reaches to 1 V threshold^39–42^. According to numerous reported studies^34–36^, the PEF with field intensity of over 0.01 V/nm on membrane structure can perforate it in few nanoseconds. So, it is reasonable to suggest the occurring of electroporation of nuclear membrane in both normal and cancerous cells. The potential difference across the nucleus in the cancerous cell is higher than that in normal cell since the cancerous cell has a larger nucleus. It correlates well to the view that cell structure with larger dimensions can be more easily electroporated as reported in literature^23^. This same view also explains that the potential difference across the cytoplasm in normal cell is higher than that in cancerous cell since the normal cell has a larger cytoplasm. Notably, the potential difference across cytoplasm is higher than that in the nucleoplasm in normal cells, while the situation is reversed in cancerous cells. This has provided a direct evidence of the selective responses of cancerous and normal cells to the short nsPEF, due to their morphological difference, which correlates well to the reported experimental observations^23^.

**Table 2:** The maximums of the electric field strength at the midpoints of each structure on the axis of two models.

The electric field strength (kV/cm)	Extracellular medium	Plasma membrane	Cytoplasm	Nucleus envelope	Nucleus cytoplasm
**Cancerous cell**					
30 ns, 50 V	2.35	1100.27	22.96	358.25	22.27
300 ns, 25 V	0.8	4593.24	9.74	301.25	10.55
500 ns, 25 V	0.78	7228.51	9.52	304.78	10.19
100 μs, 5 V	0.02	4525.42	0.26	8.68	0.23
**Normal cell**					
30 ns, 50 V	4.48	1328.20	24.91	415.11	21.50
300 ns, 25 V	1.89	5489.10	11.38	327.50	9.95
500 ns, 25 V	1.87	8374.96	11.09	326.36	9.50
100 μs, 5 V	0.22	4520.54	0.25	7.92	0.19

When the pulse width is increased to 300 ns and 500 ns, as shown in Fig. 1I-b, II-b and I-c, II-c, the potential differences across the cytoplasm and nucleoplasm in both cells rise quickly to the peak values at the beginning of the pulse, following the similar patterns in Fig. 1I-a and II-a, before starting to fall during the pulse. The peak voltage values are above 6 V and 4 V on the cytoplasm and the nucleoplasm of normal cell, respectively; while the peak voltage values are around 8 V and 3 V on the nucleoplasm and the cytoplasm of cancerous cell, respectively. At the same time, the plasma membrane in both cells is gradually charged up with a rising potential difference well above 1 V (peaking around 2.5 V in 300 ns case; and peaking around 4 V in 500 ns case) for a long period over 1200 ns, even during a long tail after the pulse, showing a long charging/discharging time constant. The potential difference on the plasma membrane is well above the electroporation threshold for the majority of the pulse duration and even the long failing tail, leading to the permeabilization of plasma membrane (cell swelling and blebbing) and possible IRE (cell necrosis)^1,3^. The nuclear envelope in both normal and cancerous cells is charged up with a rising potential difference just above 1 V for a few hundreds of ns, which again leads to the electroporation of nuclear membrane. The simulation shows that the interaction between this range of nsPEF and cells becomes complicated, involving the multiple mechanisms, i.e. a cocktail effect, though the cell necrosis caused by IRE can be dominating. Therefore, it might be difficult to correlate the cytotoxic efficiency of different cells to the cell morphology, as observed in Gianulis et al. work^19,22,24^.

As shown in Fig. 1I-d, II-d, when the pulse width is further increased to 100 μs in a conventional electroporation regime, the plasma membrane in both cells is charged up to the stable potential difference level (around 2.2 V) after a long rising edge. While the cytoplasm, nucleus and nuclear envelope in both cells can only be charged up to a low potential difference level (below 0.2 V) and then start to discharge to a very low level due to their short charging time constants as shown in Table 1. Hence, the microsecond PEF would not permeablize the membranes of intracellular compartments, but only electroporate the plasma membrane.

As shown in Fig. 1, in all exposure conditions, during the falling edge of the pulse, the potential difference on the plasma membrane falls slowly while the potential differences on the nucleoplasm, the cytoplasm and the nuclear envelop fall to negative values. It is because that the charging up time of plasma membrane is in the order of a few μs, which is much larger than those of other structures. During the falling edge of the pulse, the nucleoplasm, the cytoplasm and the nuclear envelop are reversely charged up to form negative potential differences to balance out the positive potential difference on the plasma membrane.

**Figure 2:**
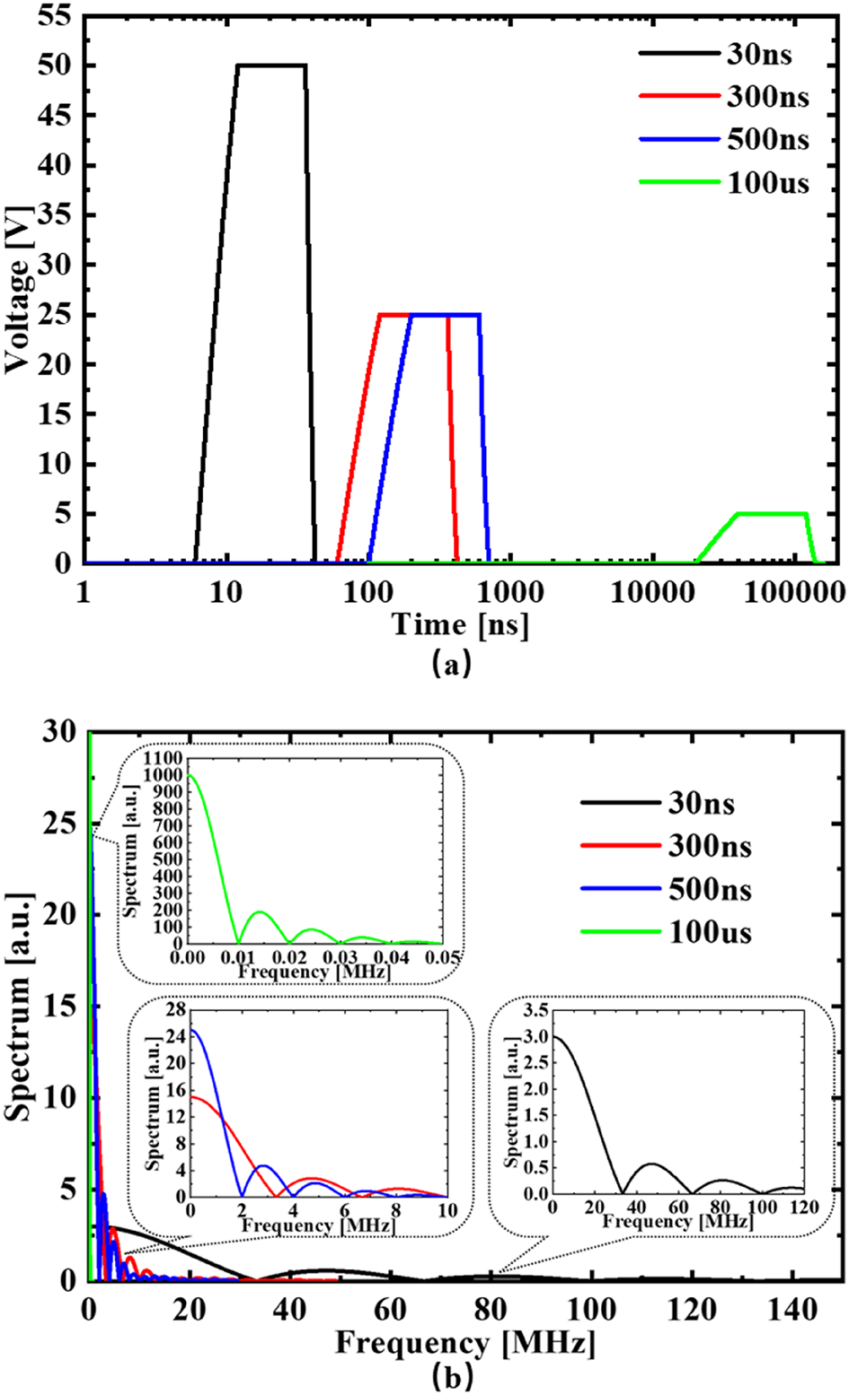
The applied voltage in (**a**) time domain and (**b**) frequency domain.

Figure 2 shows the applied voltage pulses in (a) time domain in log scale and (b) their corresponding spectra. Figure 2b shows that most of the pulse energy is in the main lobe of spectrum with 30 ns pulse main lobe stretching to 32 MHz, 300 ns pulse main lobe to 3.3 MHz, 500 ns pulse main lobe to 2 MHz and 100 µs pulse main lobe only to 0.01 MHz, respectively. So, the shorter the voltage pulse, the higher the main lobe frequency components. It is interesting to notice in Fig. 1 that the PEF with the main lobe frequency components higher than a few MHz can penetrate the plasma membrane and build up potential differences across intracellular compartments, while the PEF with low mail lobe frequency components (up to 0.01 MHz) fails. From the circuit point of view (Fig. 7c), the high capacitance plasma membrane acts like a high-pass filter to the applied PEFs. This correlates well with the reported experiments on the regulating Ca+ or inducing apoptosis using PEF, the shorter pulse width PEF with more high-frequency components can interact with the subcellular structures^47–51^.

**Figure 3:**
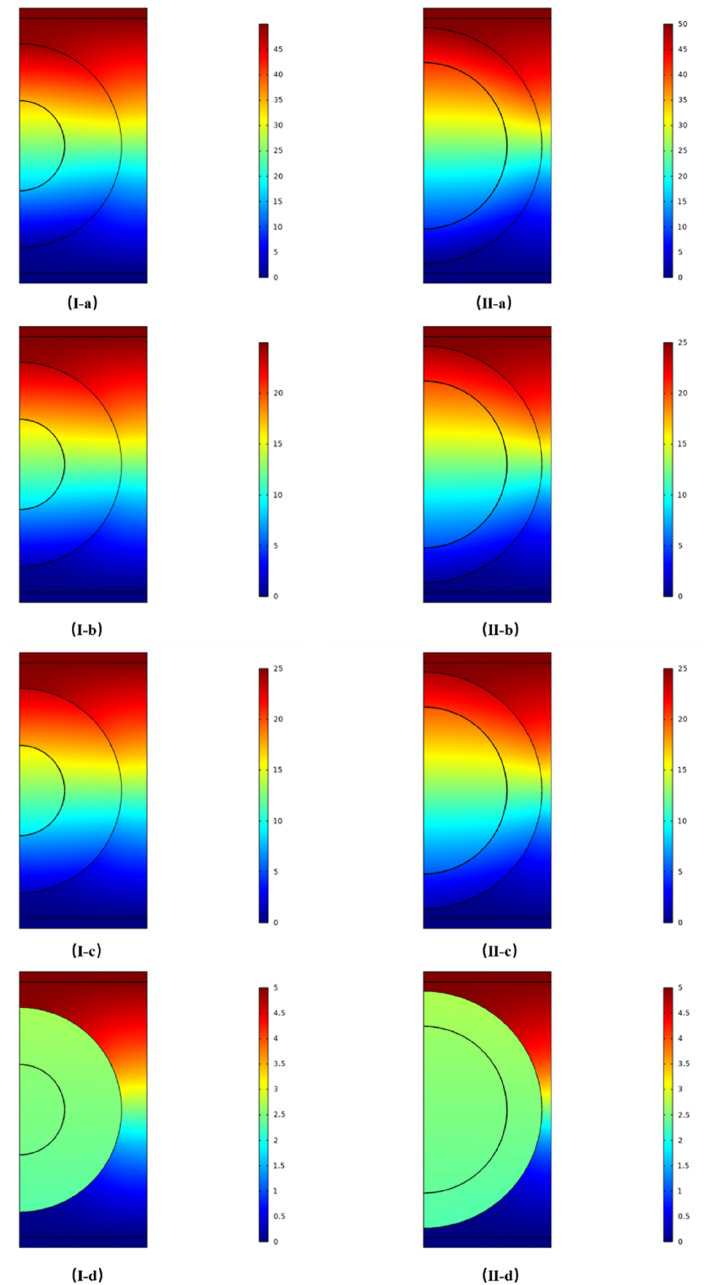
The potentials on the middle cross section of the normal (**I-a**, **b**, **c**, **d**) and cancerous (**II-a**, **b**, **c**, **d**) cell models with different time width pulses; (**I**, **II-a**): pulse width is 30 ns, voltage is 50 V, and time is 15 ns; (**I**, **II-b**): pulse width is 300 ns , voltage is 25 V, and time is 150 ns; (**I**, **II-c**): pulse width is 500 ns, voltage is 25 V, and time is 250 ns; (**I**, **II-d**): pulse width is 100 μs, voltage is 5 V, and time is 50 μs.

Figure 3 shows the potential distributions in the simulation box for the normal and cancerous cells exposed to four different voltage pulses. The electric field strength at the midpoints of each structure on the axis of models are shown in Table 2. The electric field strength in the extracellular medium along the axis is much lower, which is attributed to its high conductivity and correlates well to the negligible potential difference across it shown in Fig. 3. This can be explained using the circuit model (Fig. 7c) that a very low resistance extracellular medium doesn’t take on much voltage when connecting in series to a high resistance plasma membrane.

When the applied pulse width is 100 µs, the potential distribution is plotted at the middle of the pulse (50 µs), as shown in Fig. 3I-d, II-d. The potential inside both normal and cancerous cells is almost uniform, i.e. very low electrical field strength as shown in Table 2, which indicates that the plasma membrane has acted as a shield and prevented the penetration of the microsecond PEF into the cell. The maximum electric field strength in the plasma membrane in both cells is as high as around 4520 kV/cm as shown in Table 2, which also correlates well to the potential distribution in Fig. 3.

When the applied pulse width is shortened to a few hundred nanoseconds, the potential distributions in the middle of the pulse for 500 ns are in Fig. 3I-c and II-c, and for 300 ns are in Fig. 3I-b and II-b, respectively. The potential gradient (electric field) begins to appear inside both normal and cancerous cells, which indicates that the nanosecond PEF can easily penetrate into cells and interact with intracellular organelles as in the reported literature^12–15^. As shown in Table 2, the electric field strength in cytoplasm in normal cell is higher than that in cancerous cell, while the electric field strength in the nucleus in cancerous cell is higher than that in normal cell. These observed patterns in electric field correlate well to those patterns in potential difference in Fig. 1. The electric field strength in plasma membrane in a normal cell is higher than that in a cancerous cell, still on the level of several thousand kV/cm.

When the applied pulse width is further shortened to 30 ns, the potential distributions in the middle of the pulse are shown in Fig. 3I-a for normal cell and in Fig. 3II-a for cancerous cell, respectively. The potential gradients (electric field) inside both normal and cancerous cells look more apparent, indicating higher electric field strength as shown in Table 2. The patterns of highest electric field strength inside the normal and cancerous cells remain the same as in Fig. 3I-b and II-b. However, the potential difference across the plasma membrane can hardly been seen in Fig. 3I-d and II-d, which also correlate well to a dropped electric field strength in the plasma membrane in both normal and cancerous cells as shown in Table 2.

These observations on Fig. 3 correlate well to the evolution of potential differences across different sub-cellular structures in Fig. 1.

## Results of in-vitro experiment

**Figure 4:**
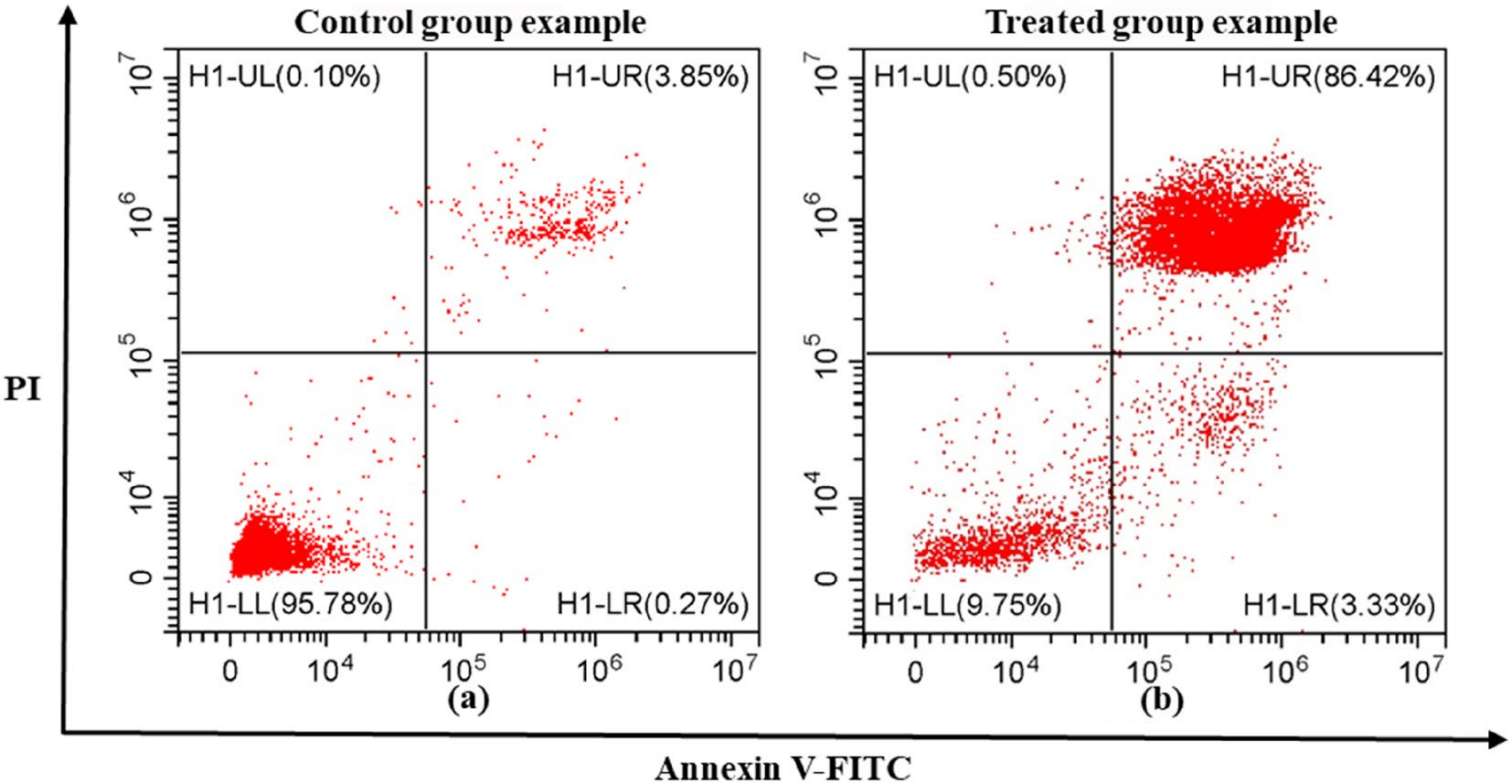
The typical fluorescence-activated cell sorting plots in the in-vitro experiments: (**a**) control group example. (**b**) Treated group example. Based on the expressions of two markers, the figures were divide into four quadrants: H1-UR represents Annexin V-FITC (+)/PI(+); H1-LL represents Annexin V-FITC (−)/PI (−); H1-LR represents Annexin V-FITC (+)/PI (−); H1-UL represents Annexin V-FITC (−)/PI (+), respectively. The data comes from the results of in-vitro experiments with L929 cells and the PEF for treated group possess the pulse width of 300 ns and the field intensity of 16 kV/cm.

The cells in control samples and nsPEF treated samples were analyzed using FACS and the typical dot plots of control groups and treated groups are shown in Fig. 4. According to the fluorescence in the control group, negative (−) or positive (+) expressions of Annexin V-FITC and PI can be defined on the diagram. Annexin V-FITC (−)/PI (−) dots represent the live cells with impermeable plasma membranes but without PS externalization; Annexin V-FITC (+)/PI (−) dots represent the early apoptosis cells with an impermeable plasma membrane and PS externalization; Annexin V-FITC (+)/PI (+) dots can represent either necrotic cells with a perforated plasma membrane or late apoptosis cells with PS externalization and high permeable plasma membrane, depending on the cell treatments^52^. Accordingly, the meanings of the four quadrants in Fig. 4 as follow: H1-UR represents the necrotic cells or the late apoptosis cells (Annexin V-FITC (+)/PI(+)); H1-LL represents the live cells (Annexin V-FITC (−)/PI (−)); H1-LR represents the early apoptosis cells (Annexin V-FITC (+)/PI (−)); H1-UL represents other cell statues (Annexin V-FITC (−)/PI (+)), respectively. In the in-vitro experiments, almost all the cells were distributed in three quadrants: Annexin V-FITC (+)/PI (+), Annexin V-FITC (−)/PI (−), and Annexin V-FITC (+)/PI (−).

It is worth mentioning that the main death mode of cells for control groups and treated groups was different. For the control groups, it is late apoptosis, and these cells have PS externalization and a highly permeable plasma membrane due to natural cell apoptotic process. For the treated group, the percentage of the cells in Annexin V-FITC (+)/PI (+) quadrant was significantly higher than that in the control group. This is because it takes several hours for cells in the early apoptosis to transfer to late apoptosis, which is much longer than the incubation time of 15 min^53,54^. The only reasonable explanation is that the plasma membrane of the treated cells was perforated by nsPEF, and Annexin V-FITC and PI crossed the perforated plasma membrane and stained PS and nuclei respectively inside the cell. Consequently, for the treated groups of cells, the main mode of death is necrosis caused by IRE, which is also indicated in the simulation.

**Figure 5:**
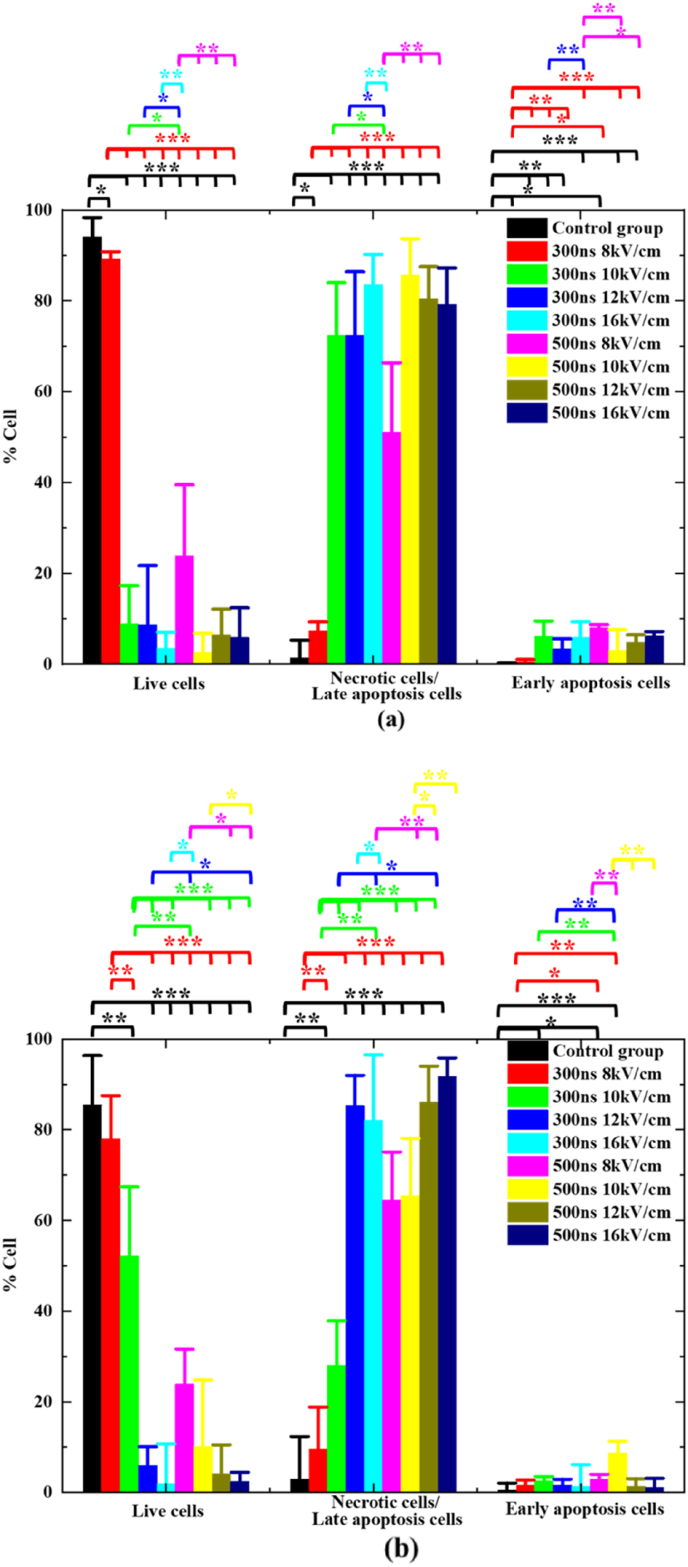
Proportions of (**a**) B16 and (**b**) L929 cells in different statuses of the cells exposed to nsPEF with different parameters and incubated for 15 min before being assayed(*P < 0.05; **P < 0.01; ***P < 0.001).

The percentages of live cells, necrotic cells/late apoptosis cells, and early apoptosis cells for B16 cells and L929 cells exposed to nsPEF with different pulse parameters are shown in Fig. 5a, b, respectively. IF50 means the electric field intensity reducing the cell survival by 50%, which can be adopted as a metric to measure cytotoxicity efficacy of nsPEF.

The results shown in Fig. 5a indicated that that the electric field intensity in B16 cells has an evident threshold effect: (1) for nsPEF with the width of 300 ns, IF50 is 8–10 kV/cm; (2) for nsPEF with the width of 500 ns, IF50 is below 8 kV/cm. Figure 5b indicated that the electric field intensity of L929 cells has evident threshold effect: (1) For nsPEF with the width of 300 ns, IF50 is 10–12 kV/cm; (2) For nsPEF with the width of 500 ns, IF50 is below 8 kV/cm. Thus, it is demonstrated that the cytotoxicity efficacy is dependent on the pulse width and intensity for both cells.

The results shown in Fig. 5 indicated that the statistical difference of the data in three quadrants are different, which part reveals the mode of death for B16 cells and L929 cells may be mixed with multiple mechanisms, including necrosis caused by IRE, and apoptosis or necroptosis caused by permeabilizing mitochondria or other intracellular organelles, which is consistent with the simulated results and previous reports^1,12–15,47^. In addition, the temperature increase caused by nsPEF was kept below 1 °C due to a low pulse repetition rate and efficient heat dissipation of the cuvette^36^. Consequently, the measured biological effects are the result of non-thermal stimulation.

**Figure 6:**
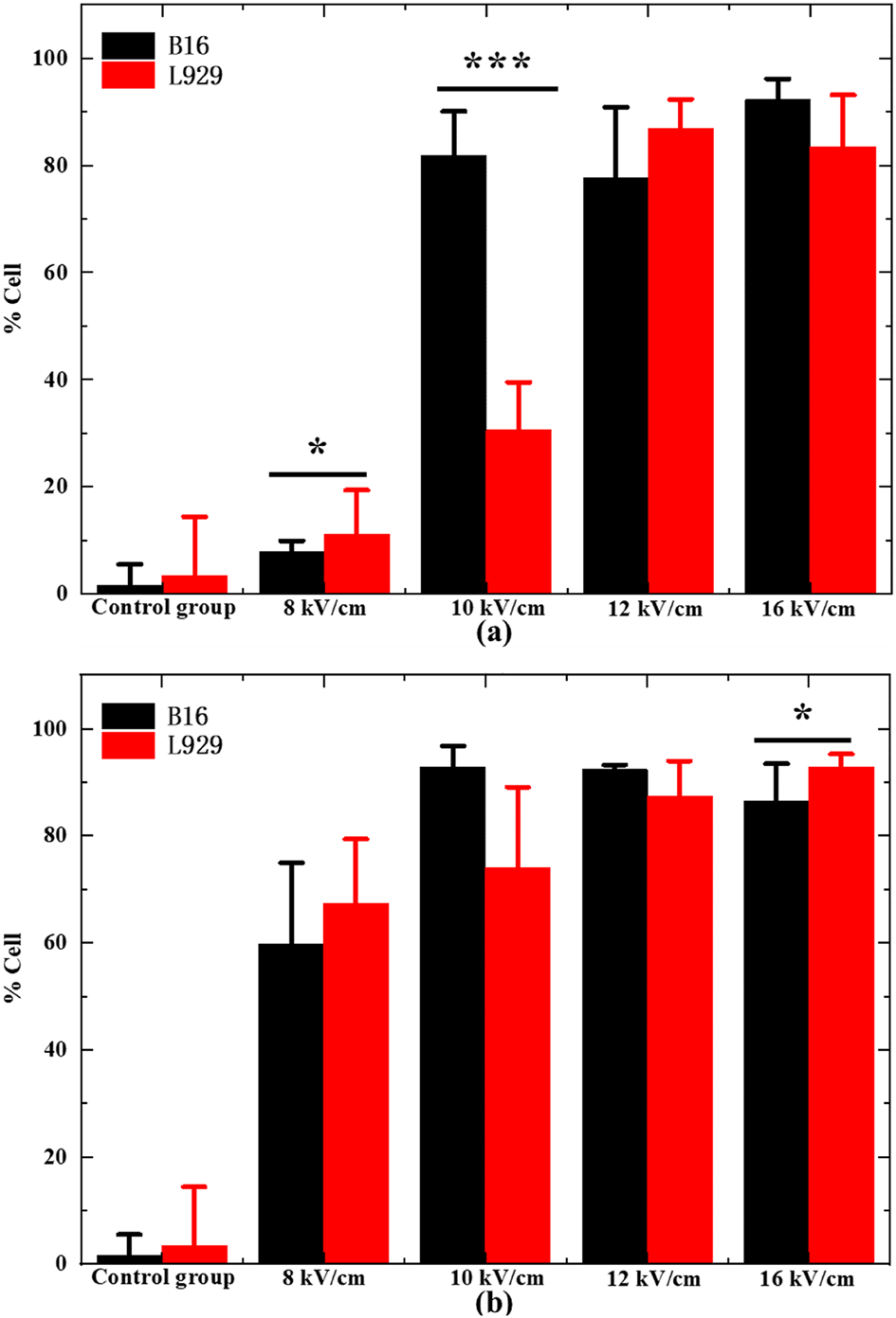
Comparison of cytotoxicity of B16 cells (black bar) and L929 cells (red bar) exposed to (**a**) 300 ns nsPEF and (**b**) 500 ns nsPEF at different intensities (8–10-12–16 kV/cm) incubated for 15 min before being assayed (*P < 0.05; ***P < 0.001).

Figure 6a, b showed the proportion of dead/necrotic cells, late and early apoptosis cells of B16 and L929 cells exposed to nsPEF with different intensity at 300 ns and 500 ns pulse width, respectively. At 300 ns pulse width as shown in Fig. 6a, a low field intensity pulse of 8 kV/cm only stimulates a small portion of cytotoxicity (< 15%) in both B16 and L929 cells. When the pulse intensity is increased to 10 kV/cm, the cytotoxicity portion increased to nearly 80% in B16 cells, but less than 30% in L929 cells. This indicated that there is a possible pulse intensity selectivity between B16 cells and L929 cells at this pulse width. When the pulse intensity is further increased to 12 kV/cm and beyond, the cytotoxicity portion fluctuates around 80% in B16 cells, but rises above 80% in L929 cells. At 500 ns pulse width as shown in Fig. 6b, a low field intensity pulse of 8 kV/cm has already stimulated a big portion of cytotoxicity (around 60%) in both B16 and L929 cells. Whilst the pulse intensity is increased to 10 kV/cm and beyond, the portion jumps above 80% in B16 cells, for L929 cells it increases steadily towards 85%. This suggested that there is no apparent selectivity between B16 cells and L929 cells. Notably, limited by the scale of the experiments, all the above results were obtained shortly after pulsing, avoiding long term effects. Long term effects of nsPEF are more complex, because of the cell division and cytokines bringing more variables^39,55^. But in our other study on the cell apoptosis, the long term effects were reported^27^.

Therefore, the in-vitro experiments have revealed that there exist the pulse width dependent threshold of electric field intensity for the cytotoxicity of normal and cancerous cells at two different pulse widths. The longer the pulse, the lower the field intensity threshold, which is consistent with previous reports^16–20^. Although the pulse width and field intensity are two coupled parameters involving complex death modes, two trends on cell toxicity related to pulse width and pulse intensity have been revealed: (1) with the same pulse width, the lower field intensity, the lower cytotoxicity; (2) with the same field intensity, the shorter pulse width, the lower cytotoxicity.

## Conclusions

The simulation study has shown that very short nsPEF (30 ns) induces a different pattern of potential difference build up on the nucleoplasm and cytoplasm between cancerous and normal cells due to their morphological difference. This correlates well to the cell selective sensitivity observed in the Yang et al. experiment^23^. When the pulse width is increased to hundreds of nanoseconds, nsPEF induces a complicated pattern of potential difference building up among the intracellular compartments in both cancerous and normal cells, indicating a mixed mode of cytotoxicity. This may explain the odd results in Gianulis et al. experiment^24^. Hence, the simulation has shown the buildup patterns of potential difference on intracellular compartments with different pulse width of PEF. For the nsPEF in the few hundreds nanosecond pulses, a complicated pattern of potential difference was built up among the intracellular compartments in both cancerous and normal cells, indicating a mixed mode of cytotoxicity (cocktail effect) and the necessity of further in-vitro experiments.

The in-vitro experiments have verified this mixed mode of cytotoxicity in the pulse range of hundreds of nanoseconds predicted by the simulation work, but also shown that the differential cytotoxicity thresholds of electric field intensity between B16 cells and L929 cells exists. The presented study provides an insight into the nsPEF interaction with the cells and a useful guide in applying nsPEF to the treatment of tumors.

## Methods

### Numerical modelling

**Figure 7:**
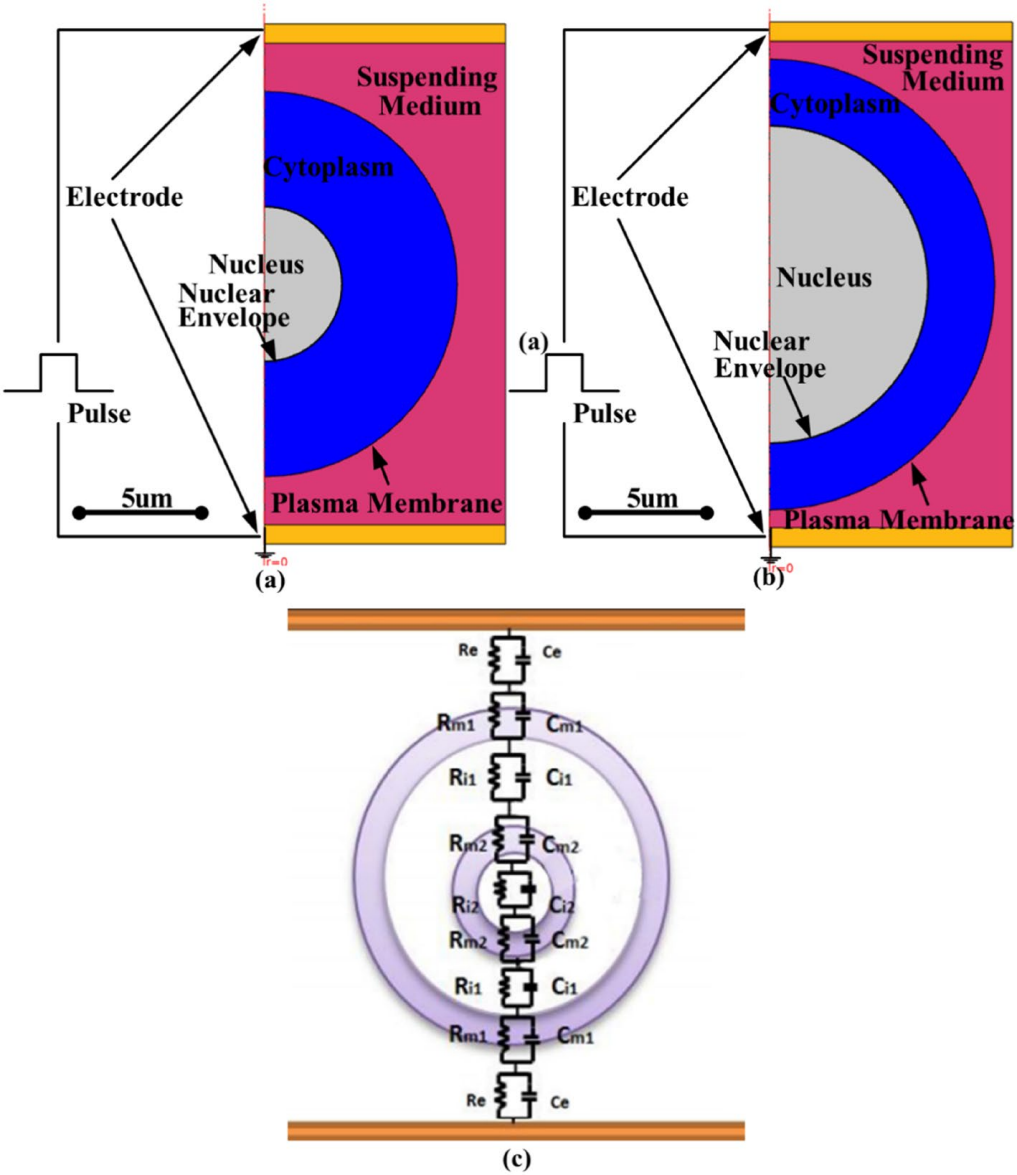
Schematic diagram of the two-dimensional rotating model of the simplified cell suspended in a conductive solution between parallel electrodes. (**a**) normal cell; (**b**) cancerous cell; (**c**) the corresponding circuit model.

To reduce the simulation scale and time, the main biological characteristics of the normal and cancerous cell were simulated based on a two-dimensional rotation model in COMSOL 5.2.1.152, a finite element time domain solver, as showed in Fig. 7a, b respectively. A pair of electrodes were placed 25 μm apart at either end of the conductive solution to apply a PEF. Like other researchers^20,56–58^ and also for the convenience of circuit analysis, a simplified spherical cell model, consisting of plasma membrane, nuclear envelope, cytoplasm and a nucleus, is set-up in the center of a conductive solution. The physical dimensions and the dielectric properties of cancerous and normal cells are taken from our measurements (Fig. S2) and the literature^20,59–62^, listed in Table 3. The rationale of these two cell models is to reflect the main morphological differences between cancerous and normal cell, instead of simulating two specific cells. The differences are the dimensions of nucleus, cytoplasm and cell diameter^59,61^. The rationale of these two cell models is to reflect the main morphological differences between cancerous and normal cell, instead of simulating two specific cells. The differences are the dimension of nucleus and cytoplasm^59,61^, which leads to different characteristic capacitance values as indicated in the corresponding circuit model shown in Fig. 7c. The characteristic capacitance of each sub-cellular structure can be estimated using the capacitance formulae for either a spherical shell or a sphere^59^. For a spherical shell,

1 $${\text{C}}_{{{\text{shell}}}} = 4\pi \varepsilon \left( {\frac{ab}{{a - b}}} \right)$$

where C_shell_ is the capacitance of the spherical shell, ɛ is the permittivity of the medium; a is the outer radius of the spherical shell, b is the inner radius of the spherical shell. For a sphere,

2 $${\text{C}}_{{{\text{sphere}}}} = 4\pi \varepsilon {\text{r}}$$

where C_sphere_ is the capacitance of the sphere, ɛ is the permittivity of the medium; r is the radius of the sphere. The calculated values of characteristic capacitance are listed in Table 1.

**Table 3:** The dielectric properties and physical dimensions of cancerous cell and normal cell^20,59–62^.

Parameters	Value (Cancerous cell)	Value (Normal cell)
Cell radius (μm)	11.59	10
Membrane thickness (nm)	5	5
Nucleus radius (μm)	8.14	4.4
Nucleus envelope thickness (nm)	40	40
Extracellular relative permittivity	80	80
Plasma membrane relative permittivity	7	7
Cytoplasm relative permittivity	60	60
Nucleus envelope relative permittivity	22.8	22.8
Nucleus cytoplasm relative permittivity	120	120
Extracellular conductivity (S/m)	1.38	1.38
Plasma membrane conductivity (S/m)	5.3E-6	5.3E-6
Cytoplasm conductivity (S/m)	0.13	0.13
Nucleus envelope conductivity (S/m)	0.0043	0.0043
Nucleus cytoplasm conductivity (S/m)	0.18	0.18

Similarly, the characteristic resistance of each sub-cellular structure can be estimated using the resistance formulae for either a spherical shell or a sphere^59^. For a spherical shell,

3 $${\text{R}}_{{{\text{shell}}}} = \frac{1}{4\pi \delta }\left( {\frac{a - b}{{ab}}} \right)$$

where R_shell_ is the resistance of the spherical shell, δ is the conductivity of the medium; a is the outer radius of the spherical shell, b is the inner radius of the spherical shell. For a sphere,

4 $${\text{R}}_{{{\text{sphere}}}} = \frac{1}{4\pi \delta r}$$

where R_sphere_ is the resistance of the sphere, δ is the conductivity of the medium; r is the radius of the sphere.

The evolution of the potential differences across four intracellular structures were observed with various pulse widths. The value of the potential difference was the potential difference of two side of each intracellular structure on the vertical central axis. The potentials on the middle cross section of models were observed at the time of half of the pulse width. The maximum electric field strength at the midpoints of each structure on the axis of models are listed in Table 3. The electric pulse with a fixed energy in extracellular solution has been assigned in the simulation with a range of pulse width: 30 ns, 300 ns, 500 ns, and 100 μs. The pulsed voltage across the top and bottom electrodes is governed by:

5 $$f({\text{t)}}\left\{ {\begin{array}{*{20}l} {{\text{A}}*(1 - e^{{ - ({\text{t}} - T_{{{\text{edge}}}} )/T_{{{\text{edge}}}} }} )} \hfill & {(T_{{{\text{edge}}}} \le t \le 2{*}T_{{{\text{edge}}}} )} \hfill \\ {{\text{A}}*(1 - e^{ - 1} )} \hfill & {(2{*}T_{{{\text{edge}}}} < t < Tpulse{ + }T_{{{\text{edge}}}} )} \hfill \\ {{\text{A}}*(e^{{ - (t - T_{pulse} - T_{{{\text{edge}}}} )/T_{{{\text{edge}}}} }} - {\text{e}}^{ - 1} )} \hfill & {(T_{pulse} { + }T_{{{\text{edge}}}} \le t \le 2{*}T_{{{\text{edge}}}} { + }Tpulse)} \hfill \\ 0 \hfill & {(else)} \hfill \\ \end{array} } \right.$$

6 $$A = 5/\left( {1 - e^{ - 1} } \right){*}\left( {11*\left( {\left\lfloor {{\text{log}}_{10}^{Tpulse} } \right\rfloor } \right)^{2} - 93*\left\lfloor {{\text{log}}_{10}^{Tpulse} } \right\rfloor + 202} \right)/12$$

where f(t) is the pulse voltage function with a variable of time t. T_*pulse*_ is the pulse width. A is a variable of T*pulse* for keeping the order of the electric field strength in line with reported literature^12–15,34–36^, and the range of the electric field strength are from 20 kV/cm to 2 kV/cm (applied voltage range is 50–5 V) for the pulse width of 30 ns to 100 μs. The rising and falling edges T_edge_ of the pulse is assigned to be 0.2* T_*pulse*_. The applied PEF in time domain and frequency domain is shown in Fig. 2.

The automatic meshing generator within COMSOL was used to generate the finite element mesh, and it consisted of 126,187 linear triangle elements. For the electrical boundary condition, electrical continuity was applied to all of the internal surfaces. An axial symmetry was applied to the left external boundary to achieve a rotation model. An electrical insulating boundary condition was applied to the right external boundary of the model, assuming that no electrical current flow through the wall of container. An electric potential was applied to the end edge of the upper electrode, which was set as the pulsed voltage as former described. A ground was applied to the end edge of the bottom electrode. The COMSOL MUMPS solver was used in this study.

### Cell tissue culture

Murine melanoma B16 cells and murine fibroblast L929 cells were obtained from Procell Life Science & Technology Co. Ltd. (Wuhan, China) and were stored frozen in liquid nitrogen until needed (the microscope images of the cells see Supplementary Fig. S2 online). They were thawed in a 37 °C water bath and then transferred to a culture flask containing RPMI-1640 (Roswell Park Memorial Institute), before being supplemented with 10% fetal bovine serum (FBS) and 2% Penicillin–Streptomycin solution. The cells were cultured in a 5% CO_2_/95% air humidified incubator at 37 °C.

### Administration of nsPEF

A cell suspension (cell concentration: 3 × 106 cells/ml, the volume: 40 μl) was loaded into the Bio-Rad cuvettes (Bio-Rad laboratories; USA) prior to nsPEF pulsing. The dielectric property of the experimental extracellular culture medium was measured using a conductivity meter (SevenCompact S230-K) and dielectric constant tester (ZJD-87), respectively. The measured conductivity is 1.38 S/m and relative permittivity is around 80. The nsPEF was delivered to the load (a 1 mm cuvette with two aluminium plate electrodes containing the cell suspension) using a self-developed digital pulse generator (the information of generator see Supplementary Fig. S1 online) with a variable pulse width of 100 ns–100 μs^25^.

After delivering the pulse, the cell suspension was removed from the pulsing cuvette and incubated for 15 min before being assayed. The exposed nanosecond pulse had a repetition rate of 2 Hz and a pulse number of 120 according to the reported work^25,37^. The independent variables were the pulse width (300, 500 ns) and the peak electric field (8, 10, 12, 16 kV/cm) as shown Table 4^25^. The control groups were standing in cuvette without exposure before incubation to reduce environmental differences as much as possible. The temperature variation in cell culture samples when exposed to 120 pulses at the repetition rate of 2 Hz at room temperature (27 °C) was continuously monitored to be less than 1 °C by using a hand-held thermograph (HIKVISON H36). All the measures in the in vitro experiments have been performed in triplicates, and all the experiments were performed in one day at room temperature (27 °C).

**Table 4:** The signal parameters of applied nsPEF in in-vitro experiments^25^.

Parameters	Value
Pulse width (ns)	300,500
Peak electric field (kV/cm)	8, 10, 12, 16
Repetition rate (Hz)	2
Pulse number	120
Rise time (ns)	150
Fall time (ns)	120
Polarity	Monopolar
Flattop decline	< 5%

### Fluorescence-activated cell sorting

In the fluorescence-activated cell sorting (FACS), the control and treated cell samples in suspension were mixed with two fluorescent markers (AnnexinV-FITC and phosphatidylserine (PI)) to determine the apoptotic related death mode of cells^63,64^. The first marker was phosphatidylserine (PS) externalization as indicated by Annexin V-FITC binding to intact plasma membranes, which emits green fluorescence with the excitation of blue light. The second marker was plasma membrane permeability as indicated by transmembrane PI binding to DNA stain, which emits red fluorescence. The cell samples were subjected to flow cytometry using a Beckman Coulter Flow cytometer (Danaher Corporation; USA). At the same time, cells in each sample were excited with a 488-nm (blue light) argon laser and sorted according to their fluorescence expressions to the AnnexinV-FITC (green light) and the PI (red light) after an initial screening of cell integrity. Then, the sorted cells were plotted as scattering dots on a 2D diagram using the degrees of these two marker expressions as the coordinates, as shown in Fig. 4. The FACS test for each sample accomplished in few minutes to avoid the influence of cytotoxicity of staining.

### Statistics

All data and analysis of variance were processed with the self-developed Matlab program based on the T-test algorithm. The critical values were set at *P* < 0.05/0.01/0.001.

## Supplementary Information

Supplementary Information.

## Data Availability

All data generated or analysed during this study are included in this published article and its supplementary information files.
