# Supplementary material for: Pulse width and intensity effects of pulsed electric fields on cancerous and normal skin cells
Source: Sci Rep. 2022 Oct 27;12:18039. doi: 10.1038/s41598-022-22874-x (PMC9613658; doi:10.1038/s41598-022-22874-x)
Supplement: Supplementary file 1 — Supplementary Information. [file 41598_2022_22874_MOESM1_ESM.docx]

**Pulse Width and Intensity Effects of Pulsed Electric Fields on Cancerous and Normal Skin Cells**

**Supplementary materials**

The pulse generator system is implemented in the Lab, as shown in Figure S1 (a). The measured voltage pulses on the cuvette and their spectra are shown in Figure S1(b) and (c), respectively. Comparing Figure S1 (b), (C) and Figure 2, the waveforms of the applied voltage and simulated voltage are similar.

**
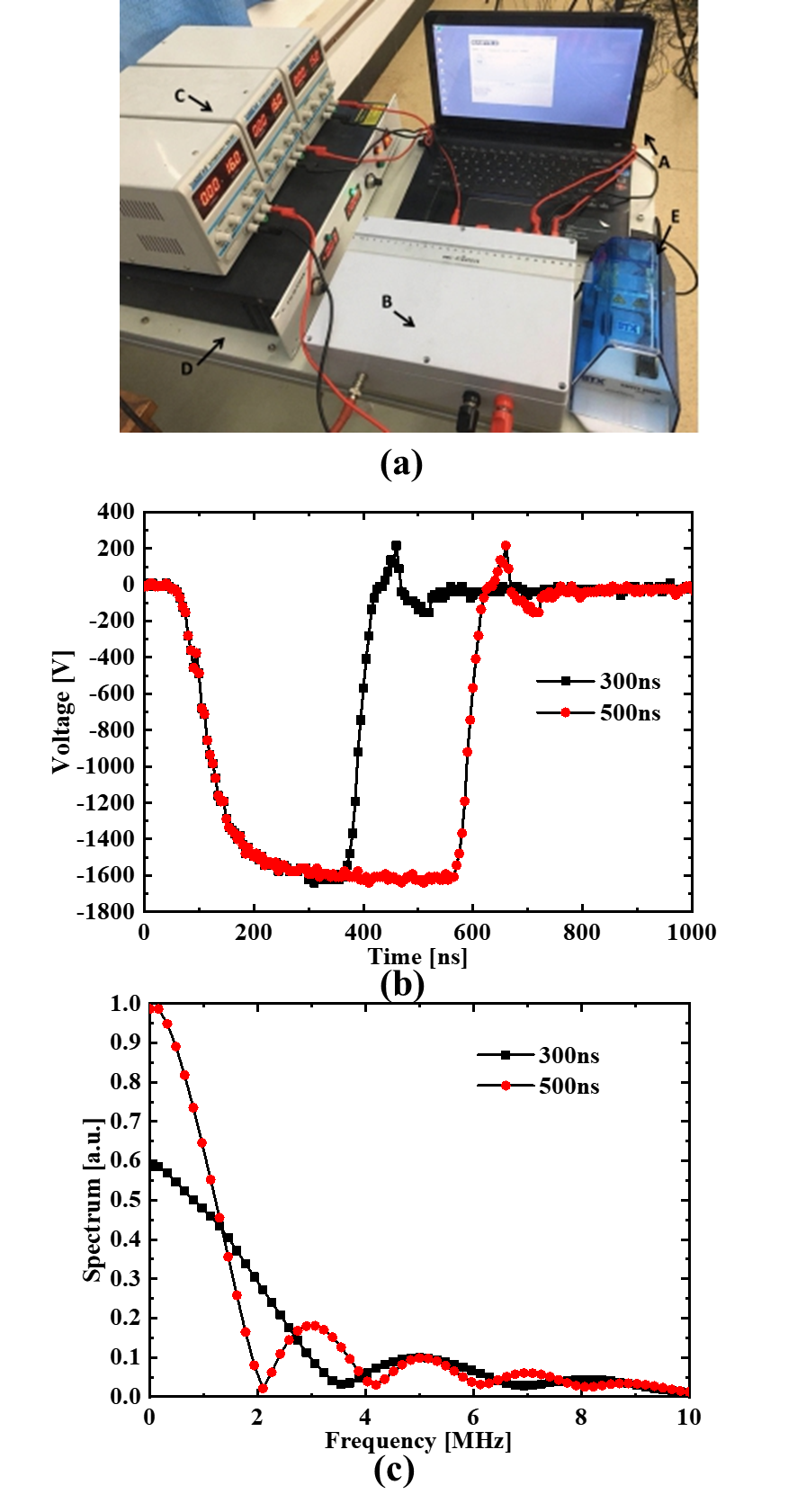
**

**Figure S1.** The information of generator: (a) The photo of the pulse generator system and the waveform of the 300/500 ns and 16 kV in (b) pulses in time domain and (c) pulses in frequency domain.

The cell were fixed with 4% paraformaldehyde for 15 min, then were centrifuged for 5 min, finally were washed and resuspended with PBS. The photo were taken with smear microscopy. As Figure S2 shown, the cells are nearly spherical, and the difference in morphology of two kinds of cells can be easily observed. The measured cell dimensions are: B16: 18.1-25 µm, L929: 17.74-24.38 µm, respectively.

**
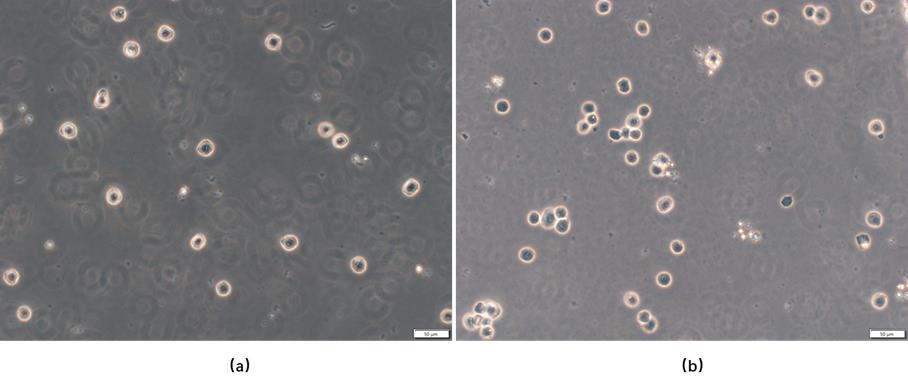
**

**Figure S2.** The microscope images (X200) of (a) L929 cells and (b) B16 cells.
